# Reconstructive Osteotomy for Ankle Malunion Improves Patient Satisfaction and Function

**DOI:** 10.1155/2015/549109

**Published:** 2015-04-29

**Authors:** Fumiaki Inori, Masahiko Tohyama, Hiroyuki Yasuda, Sadahiko Konishi, Akeo Waseda

**Affiliations:** ^1^Department of Orthopedic Surgery, Osaka General Hospital, West Japan Railway Company, Japan; ^2^Department of Orthopedic Surgery, Ogikubo Hospital, Japan

## Abstract

Treatment of chronic symptoms caused by a malunion is a difficult problem in orthopedic surgery. We encountered a case of ankle malunion at our hospital about 1 year after the first operation. The patient had been unable to walk with weight-bearing but regained the ability to walk after reconstructive osteotomy of the fibula. Functional scores for the foot and ankle were significantly improved after intervention. Reconstructive osteotomy appears to represent a good option for ankle malunion.

## 1. Introduction

Ankle malleolar fracture is a common bone fracture. If the degree of displacement is large, open reduction and internal fixation (ORIF) should be performed. However, if anatomical reduction is insufficient and fixation is inadequate, malunion might result, damaging ankle function. If such ankle malunion occurs, reconstructive osteotomy, arthrodesis, or artificial arthroplasty can be considered. We present here a case of ankle malunion in which ankle joint function was restored by reconstructive osteotomy despite intervention occurring 1 year after the first ORIF.

## 2. Case Report

A 44-year-old woman injured her right ankle joint after falling from a bicycle in January 2013. One week later, ORIF was performed at the initial emergency hospital. One month postoperatively, the patient was permitted to walk with weight-bearing but was unable to do so due to sustained ankle pain on the lateral and posterior aspect. After follow-up, including rehabilitation for 7 months, all screws were removed under the assumption that bone union had been obtained. However, ankle pain remained and the patient still could not walk with weight-bearing and was referred to our hospital in December 2013.

**Figure 1:**
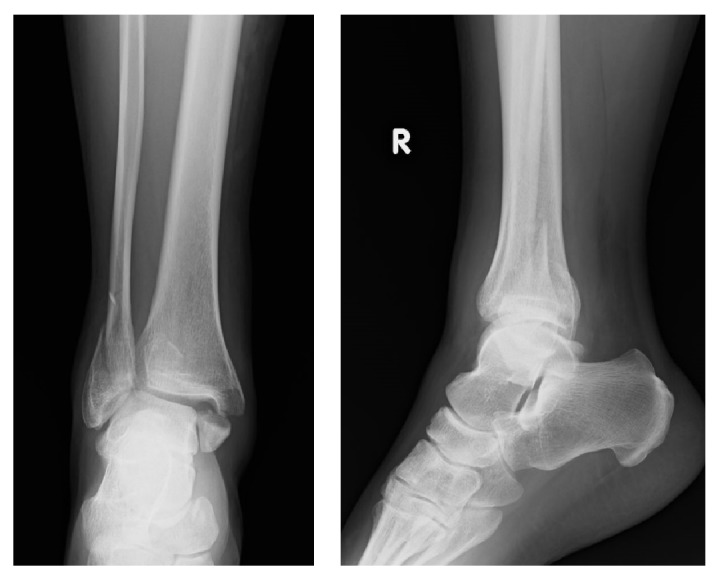
Initial radiographs at emergency hospital. Weber type B and LH classification's SE type stage 4 fractures.

**Figure 2:**
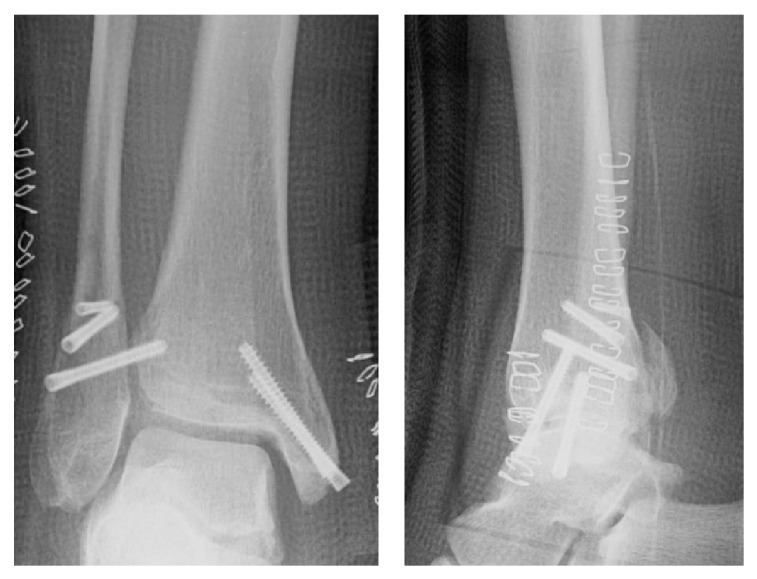
Radiographs soon after the 1st operation. Medial and lateral malleolus were fixed by Acutrak 2 screws.

**Figure 3:**
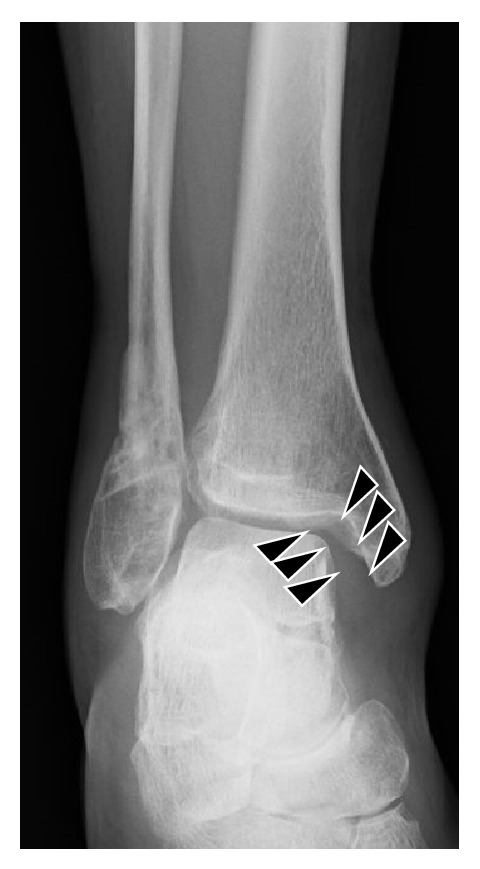
Radiographs at the time of admission to our hospital, after the removal of screws. A shortening of the fibula relative to the medial malleolus, widening of the ankle fork, and a valgus tilt of the talus causing an abnormally widened medial clear space (black triangle).

**Figure 4:**
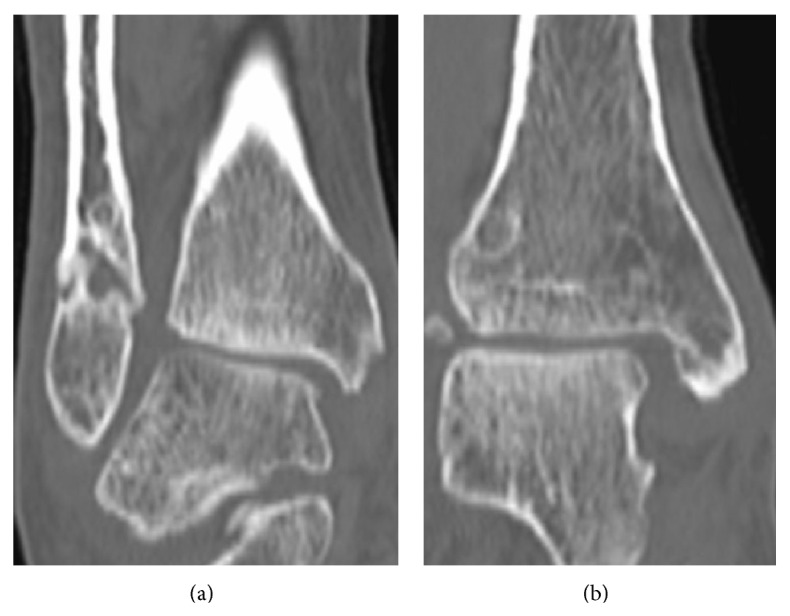
CT coronal view at the time of admission to our hospital. (a) Lateral malleolus was malunited, (b) in spite of medial malleolus being united.

On initial plain radiographs, Weber type B and Lauge-Hansen classification SE type stage 4 fractures were revealed (Figure 1). The medial and lateral malleoli were fixed using Acutrak 2 screws (Acumed, Hillsboro, OR) at the time of initial operation in February 2013 (Figure 2). After the removal of those screws, a medial clear space was opened (Figure 3). Computed tomography at our hospital revealed bone union of the posterior and medial malleoli but malunion of the lateral malleolus (Figure 4).

Ankle arthrodesis is considered one form of salvage when a long time has elapsed since the first operation. However, reconstructive osteotomy was selected in this case because of the young age of the patient and the relatively smooth joint surface on radiography. In this case, shortening of the fibula because of posterolateral rotation of the distal fragment and widening of the ankle fork because of concomitant syndesmotic injury was present. As a result, the surgical plan involved fibular osteotomy through the initial fracture plane with fibular lengthening and internal rotation of the distal fragment until the ankle fork had been reconstructed anatomically. In addition, syndesmotic stabilization was planned by screw fixation.

**Figure 5:**
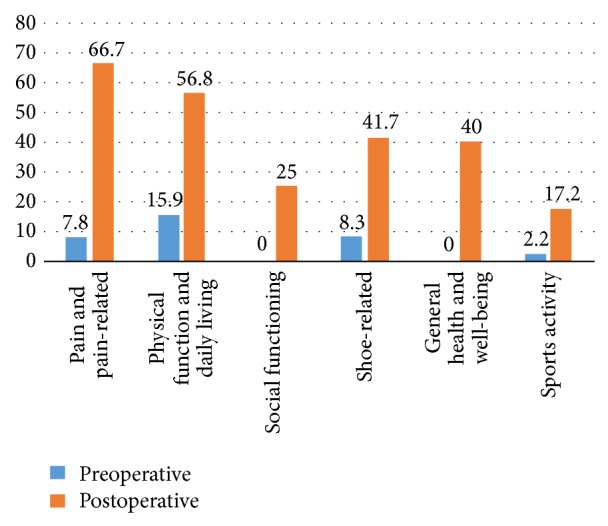
Pre- and postoperative (5 months after operation) evaluated foot score of SAFE-Q. The score was significantly improved in all subscale categories.

We also evaluated preoperative foot function before reconstructive osteotomy using the self-administered foot evaluation questionnaire (SAFE-Q) (Figure 5) [1]. Despite poor results, ankle range of motion was relatively well maintained from 10° of dorsiflexion to 30° of plantar flexion.

**Figure 6:**
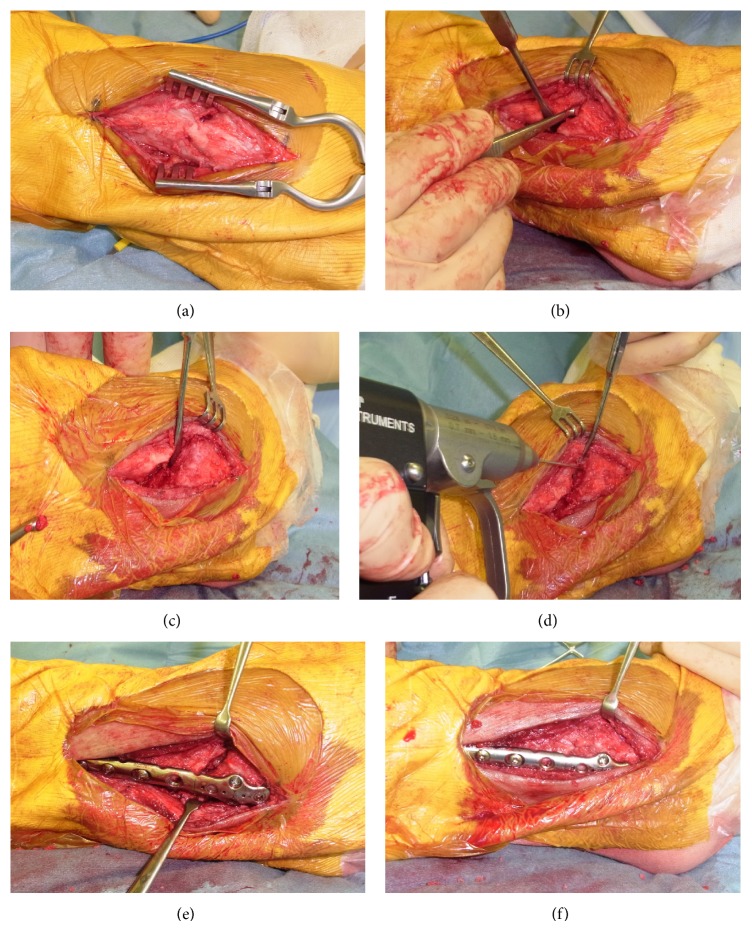
Operative procedure of lateral malleolus. (a) Malunion part of fibula was exposed. (b) Scar tissue was removed. (c) Partial united bone was recut with chisel, and distal and proximal bone fragments were separated completely. (d) Corticated part of bone was decorticated with 1.2 mm K-wire. (e) The bone fragments were reconstructed as close as possible to normal side under the fluoroscopic image; then bone gap was arisen as shown. (f) Autologous iliac cancellous bone was grafted into this gap and bone fragments were fixed with locking plate.

Revision was performed in February 2014, almost 1 year after the first operation. At first, the malunited part of the fibula was exposed and scar tissue was removed. Partially united bone was recut with a chisel, and distal and proximal bone fragments were completely separated. Corticated surface of bone fragments was decorticated with a 1.2 mm K-wire. The bone fragments were reconstructed as close as possible to the normal side under fluoroscopic imaging; then a bone gap was created (Figure 6). Autologous iliac cancellous bone was grafted into this gap and bone fragments were fixed with a locking plate. Next, a syndesmotic compression screw was inserted to fix the tibiofibular joint. Confirming the medial side, the medial triangle ligaments were loose and were therefore corrected by resuturing firmly. The operating time was 2 h 36 min, and blood loss was minimal.

**Figure 7:**
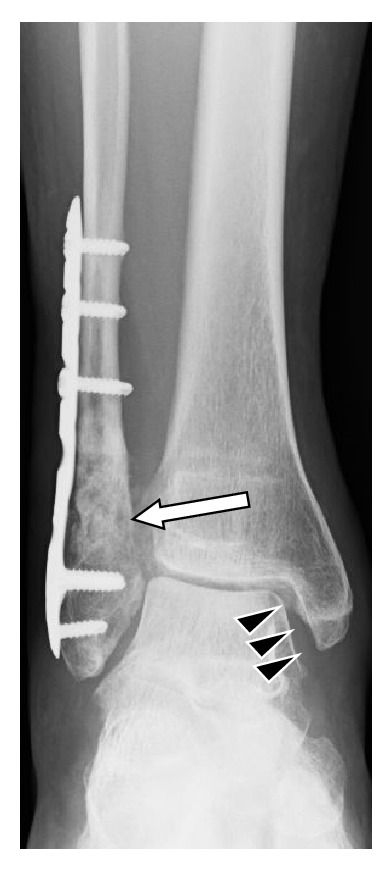
Radiograph 5 months after the 2nd operation. All abnormalities displayed in Figure 3 have been reconstructed to normal with the treatment that has been performed. Concerning fibula, grafted bone was not absorbed and alignment was not changed after second operation (white arrow). This properly aligned fibula, tightened ankle fork, and restored valgus tilt of the talus maintained medial clear space properly (black triangle).

Postoperatively, a short leg cast was applied and maintained for 4 weeks and then changed to a half cast and range of motion exercises for the ankle were started. The compression screw was removed at 6 weeks postoperatively, and partial weight-bearing walking was started. Full weight-bearing with an ankle support was achieved 12 weeks postoperatively. Radiography after 5 months revealed that the medial clear space had been properly maintained. With regard to the fibula, grafted bone was not absorbed and alignment was unchanged after the second operation; bone union was considered to have been achieved (Figure 7).

SAFE-Q was also administered at 5 months postoperatively, showing significant improvements in all subscales (Figure 5). Postoperative range of motion was 10° of dorsiflexion and 40° of plantar flexion. Compared with the range of motion before the second operation, little change was evident.

## 3. Discussion

The treatment of ankle malunion is controversial. Reconstructive osteotomy has been reported to show good or excellent results in more than 75% of patients if performed before arthritic changes develop, according to a systematic review [2]. The period between onset and operation has also been reported to bear no relation to the result, whereas correction as soon as possible before osteoarthritic changes developed was recommended [3–5]. In our case, almost 1 year had passed from the initial operation, but radiographic osteoarthritic changes had not appeared, so reconstructive osteotomy was selected and led to good results.

Similarly, untreated bimalleolar malunion has been successfully treated using bimalleolar reconstructive osteotomy more than 8 months from the initial injury [6]. In that case, the medial malleolus was corrected first due to severe angular deformity, followed by correction of the lateral malleolus. Proper anatomical alignment and stable fixation were considered keys to the successful result for that patient.

Fortunately, the medial malleolus was not severely deformed in our case, and correction of the lateral malleolus alone allowed restoration of lateral talar displacement. Recutting the original fracture site and restoring comparable length and rotation to the contralateral healthy side were key factors.

SAFE-Q score was substantially improved after surgery, especially in the pain and functional categories (Figure 5). SAFE-Q is a quality-of-life questionnaire instrument for use in evaluating pathological conditions related to the foot and ankle and was developed by the Japanese Society for Surgery of the Foot [1]. This instrument consists of 34 questionnaire items, providing five subscale scores ((1) pain and pain-related; (2) physical functioning and daily living; (3) social functioning; (4) shoe-related; and (5) general health and well-being). In this case, significant improvements were achieved in all subscales (Figure 5). We confirmed that reconstructive osteotomy affects not only ankle function but also patient satisfaction.

According to the literature, only advanced degenerative changes have been considered as contraindications for reconstructive osteotomy [3, 5, 7–12]. Arthrodesis and arthroplasty should be reserved as salvage alternatives for progressive deformity or failed cases.

In conclusion, reconstructive osteotomy for ankle malunion is effective even if more than 1 year has passed since the first operation. Ankle function and patient satisfaction were significantly improved by this procedure.

## References

[1] Niki H., Tatsunami S., Haraguchi N. (2013). Validity and reliability of a self-administered foot evaluation questionnaire (SAFE-Q). *Journal of Orthopaedic Science*.

[2] van Wensen R. J. A., van den Bekerom M. P. J., Marti R. K., van Heerwaarden R. J. (2011). Reconstructive osteotomy of fibular malunion: review of the literature. *Strategies in Trauma & Limb Reconstruction*.

[3] Yablon I. G., Leach R. E. (1989). Reconstruction of malunited fractures of the lateral malleolus. *The Journal of Bone and Joint Surgery. American Volume*.

[4] Chao K.-H., Wu C.-C., Lee C.-H., Chu C.-M., Wu S.-S. (2004). Corrective-elongation osteotomy without bone graft for old ankle fracture with residual diastasis. *Foot and Ankle International*.

[5] Roukis T. S. (2004). Corrective ankle osteotomies. *Clinics in Podiatric Medicine and Surgery*.

[6] Robertson J., Alexander K. (2011). Delayed reconstruction of post traumatic ankle malunion: a case report. *The Foot and Ankle Online Journal*.

[7] Henderson W. B., Lau J. T. C. (2006). Reconstruction of failed ankle fractures. *Foot and Ankle Clinics*.

[8] Weber B. G., Simpson L. A. (1985). Corrective lengthening osteotomy of the fibula. *Clinical Orthopaedics and Related Research*.

[9] Offierski C. M., Graham J. D., Hall J. H., Harris W. R., Schatzker J. L. (1982). Late revision of fibular malunion in ankle fractures. *Clinical Orthopaedics and Related Research*.

[10] Weber B. G. (1981). Lengthening osteotomy of the fibula to correct a widened mortice of the ankle after fracture. *International Orthopaedics*.

[11] Davis J. L., Giacopelli J. A. (1995). Transfibular osteotomy in the correction of ankle joint incongruity. *Journal of Foot and Ankle Surgery*.

[12] Miller S. D. (1995). Late reconstruction after failed treatment for ankle fractures. *Orthopedic Clinics of North America*.

